# Role of Artificial Intelligence in Surgical Decision-Making: A Comprehensive Review

**DOI:** 10.31661/gmj.v13i.3332

**Published:** 2024-03-19

**Authors:** Anita Zarghami

**Affiliations:** ^1^ Department of Surgery, Shahid Beheshti University of Medical Sciences, Tehran, Iran

**Keywords:** Artificial Intelligence, Surgical Decision-Making, Outcome, Electronic Health Record, Cancer

## Abstract

Artificial intelligence (AI) has emerged as a promising technology that can revolutionize surgical decision-making (SDM). This comprehensive review aims to explore the current state of AI in SDM and highlight its benefits, challenges, and future directions. The integration of AI in SDM offers numerous advantages. AI algorithms can analyze medical images, such as radiographs, computed tomography scans, and magnetic resonance imaging, to detect abnormalities and assist in pre-operative assessments. By leveraging electronic health records, AI can provide personalized surgical recommendations based on patient-specific data.Additionally, AI can analyze genetic data to assess genetic predispositions and tailor treatment plans accordingly. Intra-operatively, AI can aid in real-time analysis of surgical videos and imaging, helping surgeons identify critical structures and guide precise incisions. AI algorithms can also monitor physiological indicators to detect early signs of complications and predict outcomes, improving intra-operative decision-making. Post-operatively, AI can analyze vital signs, imaging, and patient data to detect complications, provide outcomes analysis, and facilitate personalized patient care. However, challenges and limitations exist. Data quality and availability, interpretability of AI algorithms, data security, integration into surgical workflows, and regulatory considerations are important challenges. Addressing these challenges involves ensuring data privacy, developing transparent AI models, establishing robust infrastructure, engaging clinicians, and establishing regulatory frameworks. AI-powered surgical robots and systems can enhance surgical precision and automation. Improvements in interpretability and explainability foster trust and ethical considerations. Also, data sharing and collaboration advancements could refine AI algorithms’ accuracy and generalizability. Personalized medicine and precision surgery are achieved through AI integration. Also, education and training could benefit from AI-powered decision support systems.

## Introduction

Surgical decision-making (SDM) is a complex and critical process that requires careful analysis, extensive knowledge, and years of experience [1]. It involves various factors, including patient history, symptoms, imaging results, and surgical techniques, to determine the most appropriate course of action [2]. The integration of artificial intelligence (AI) technology into healthcare has sparked significant interest in exploring its potential role in enhancing SDM [3][4]. Recent advances in AI, such as machine learning, deep learning, and expert systems, have shown great promise in revolutionizing medicine [5]. AI algorithms could analyze vast amounts of data quickly and accurately, providing valuable insights and predictions that can significantly improve surgical outcomes [6]. By leveraging these technologies, surgeons can make more informed decisions, enhance surgical precision, reduce complications, and improve patient care. Hence, this comprehensive review aims to provide the current state-of-the-art applications of AI in SDM. Also, its roles in pre-operative assessments, intra-operative decision-making, and post-operative monitoring were reviewed.

## AI in Pre-operative Assessments

AI has shown great potential in revolutionizing pre-operative assessments, which are crucial in determining the optimal surgical approach and predicting patient outcomes [7]. One of the key applications of AI in this area is the analysis of medical images, such as radiographs, computed tomography (CT) scans, and magnetic resonance imaging (MRI) scans [8][9]. Indeed, machine learning algorithms can rapidly analyze these images, identifying patterns and anomalies that may be missed by routine evaluations [10]. This feature enables accurate detection of diseases, tumors, or other abnormalities that may impact the SDM process.

Furthermore, AI can use electronic health records (EHRs) to provide personalized surgical recommendations [11]. Indeed, AI algorithms could identify relevant factors that impact surgical outcomes by processing extensive patient-specific data, such as medical history, lab results, medications, and demographic information [12]. Hence, this capability allows surgeons to make more informed decisions by considering the patient’s characteristics and tailoring treatment plans accordingly [13].

In addition to medical images and EHRs, AI can analyze genetic data to assist in pre-operative assessments. By examining a patient’s genetic profile, including DNA sequencing data or specific markers, AI algorithms can identify genetic predispositions or markers associated with certain diseases and/or treatment responses [14]. Hence, this valuable information helps surgeons evaluate the risks and benefits of specific surgical interventions, leading to more precise and personalized approaches [15].

The integration of AI in pre-operative assessments offers several benefits. It could enhance the accuracy and efficiency of diagnoses, as AI algorithms can evaluate medical images and data rapidly and, in some cases, even surpass human performance [16][17]. This efficiency could reduce the time and effort required for assessments and ensure timely and accurate diagnosis, which leads to improved patient outcomes. Moreover, AI in pre-operative assessments can provide valuable insights into the likelihood of surgical success and potential complications [18]. Also, by analyzing large datasets and identifying patterns, AI algorithms can predict the probability of positive outcomes or post-operative complications [19].

## AI in Intra-operative Decision-making

AI technology can potentially play a transformative role in intra-operative decision-making-a critical phase of the surgical process-where real-time analysis and immediate actions are required [20]. AI algorithms can process and analyze data from various sources, such as surgical instruments, imaging, and physiological indicators to provide valuable insights and support for surgeons [21]. One another key application of AI in intra-operative decision-making is the real-time analysis of surgical videos and images [22]. Indeed, analyzing visual data by AI algorithms can assist surgeons in identifying critical anatomical structures, guiding precise incisions, and accurately removing tumors or lesions [23]. In addition, AI can integrate information from different imaging modalities, such as ultrasound, MRI, or CT scans, to provide surgeons with a comprehensive and multidimensional view of the surgical site [23][24]. This data fusion allows for enhanced visualization and planning, facilitating more informed decision-making during the surgical procedure. Additionally, AI algorithms can process physiological indicators, which are obtained from monitoring devices, such as heart rate, blood pressure, and oxygen saturation levels [25]. Moreover, AI can assist in predicting potential complications or adverse events during surgery [26]. Also, AI algorithms can provide risk assessments and outcome predictions by analyzing large datasets and considering multiple variables, including patient characteristics, surgical techniques, and historical data [27]. This predictive capability helps surgeons anticipate possible challenges, optimize their surgical strategies, and improve patient safety and outcomes.

The integration of AI in intra-operative decision-making has several advantages. It provides real-time, objective, and data-driven guidance to surgeons to reduce the risk of human error and enhance outcomes. Indeed, by combining surgeons’ expertise with AI’s analytical ability, surgical procedures can become safer, more precise, and more effective, ultimately improving patient outcomes [30]

## AI in Post-operative Monitoring and Analysis

AI has emerged as a valuable tool in post-operative monitoring and analysis, contributing to enhanced patient care, early detection of complications, and improved surgical outcomes [31]. By continuously monitoring and analyzing patient data, AI algorithms could provide valuable insights and support to healthcare providers during the crucial post-operative phase [32]. One important application of AI in post-operative monitoring is the analysis of vital signs and laboratory values [33]. AI algorithms can process real-time data (e.g., heart rate, blood pressure, temperature, or oxygen saturation levels) and alert healthcare providers to any abnormal changes and potential complications [34]. This enables timely intervention and proactive management, reducing the risk of adverse events and improving patient safety.

AI can also analyze post-operative imaging, such as X-rays, CT scans, or MRIs, to detect and monitor post-surgical changes or complications [35][36]. By evaluating and comparing these images to pre-operative imaging, AI algorithms can identify any abnormalities, such as signs of infection, bleeding, and organ dysfunction [37]. This rapid analysis aids in early detection and prompt intervention, leading to improved patient outcomes. Furthermore, AI can analyze post-operative data to identify trends and patterns associated with surgical success or failure [38].

Also, AI assists in outcomes analysis, facilitating evaluating and comparing different treatment strategies and surgical techniques [39]. AI algorithms can identify factors influencing surgical outcomes, such as length of hospital stay, post-operative complications, or re-admission rates [40]. This information can help to enhance quality improvement, refine surgical protocols, and optimize patient care.

Alongside monitoring and analysis, AI can automate post-operative follow-up and patient communication [41]. Through chatbots or virtual assistants, AI technology can provide patients with personalized and evidence-based guidance on post-operative care, medication management, and potential adverse effects [42]. As a result, this support and continuous monitoring can alleviate patient anxieties, improve adherence to post-operative instructions, and enhance patient satisfaction [43].

## Challenges and Limitations

The integration of AI in SDM brings various challenges and limitations that must be addressed to ensure the safe and effective implementation of AI technologies in surgical practice [44]. One primary concern is the quality and availability of data. AI algorithms rely on high-quality data to train and produce accurate predictions or recommendations [45]. However, there may be challenges in accessing comprehensive and representative datasets, particularly for rare conditions or specific patient populations [46]. Biases within the data, such as demographic disparities or limited diversity, can also impact the generalizability and fairness of AI algorithms [47].

Another significant challenge is the interpretability and explainability of AI algorithms [48]. Many AI approaches, such as deep learning, operate as black boxes, making it difficult to understand how the algorithms arrive at particular predictions and/or decisions [49][50]. This lack of interpretability raises concerns about trust, accountability, and ethical considerations. It is crucial to develop AI models that provide transparent explanations to users, enabling clinicians to understand and trust their recommendations [51]. Data security and privacy are additional challenges that cannot be overlooked [52]. Using sensitive patient data in AI algorithms requires robust privacy protections. Healthcare organizations must establish secure infrastructures, implement rigorous data anonymization techniques, and ensure compliance with privacy regulations to maintain patient confidentiality and protect against data breaches [53].

Integration and acceptance of AI algorithms within surgical workflows pose further challenges [54]. Surgeons and healthcare professionals may be resistant to adopting AI technologies due to concerns about job displacement, loss of professional autonomy, or lack of trust in AI’s capabilities [55]. It is essential to actively engage surgeons in the development and validation process and build trust and collaboration to ensure the successful integration of AI into SDM [56].

Regulatory considerations also emerge as a significant challenge. AI algorithms used in SDM should adhere to regulatory guidelines to ensure patient safety and efficacy [57]. Indeed, developing regulatory frameworks that could accommodate the dynamic and rapidly evolving nature of AI technologies is essential to ensure patient safety without stifling innovation or hindering progress [58]. Moreover, the validation and robustness of AI algorithms in real-world clinical settings remain an ongoing challenge [59]. Indeed, AI systems must demonstrate high accuracy, reliability, and generalizability before being adopted into routine surgical practice [60]. Hence, various validation studies among different patient populations and surgical specialties are necessary to ensure the efficacy and safety of AI algorithms [60][61].

Despite these challenges, it is important to recognize that AI technologies in SDM have immense potential to revolutionize patient care [61]. Addressing these challenges requires interdisciplinary collaboration among clinicians, AI researchers, regulatory bodies, and industry stakeholders [62]. Ethical frameworks, guidelines, and standards must be developed to ensure AI’s responsible and ethical use in SDM, ultimately advancing the field and benefiting patients worldwide [63].

## Future Directions

The future of AI in SDM is promising, with several important directions for further advancements. As technology continues to evolve, there are several key areas where AI can significantly improve surgical outcomes and patient care [64].

One direction involves the development of AI-powered surgical robots and systems [65]. These robots can assist surgeons in performing complex procedures with greater precision and dexterity [66]. AI algorithms can enable real-time feedback and guidance, allowing surgeons to navigate challenging anatomical structures and perform minimally invasive surgeries with enhanced accuracy [67]. The integration of machine learning and computer vision can aid in automating specific surgical tasks, reducing surgical time, and improving patient recovery [68].

Another important future direction is to increase the ability to intercept and explain AI models [69]. As AI algorithms become more complex, it becomes increasingly essential to understand the underlying logic behind their decisions [70]. Research efforts are focused on developing AI models that can provide transparent explanations and greater confidence and trust in incorporating AI recommendations into their SDM by both surgeons and healthcare professionals [71][72]. Explainable AI is crucial for ethical considerations, regulatory compliance, and patient safety.

In addition, advancements in data sharing and collaboration play a vital role in the future of AI in SDM [73]. By securely sharing anonymized patient data across healthcare institutions and research organizations, AI algorithms can be trained on more extensive and diverse datasets, leading to improved accuracy and generalizability [74]. Collaborative efforts can facilitate the development of robust AI models that account for variations in surgical techniques, patient populations, and healthcare systems [75].

Furthermore, AI integration could significantly progress personalized medicine and precision surgery [76]. AI algorithms can analyze diverse patient characteristics, such as genetics, biomarkers, and medical history, to create customized treatment plans, predict individual outcomes, and tailor surgical strategies accordingly [77]. AI can assist in identifying optimal surgical techniques, predicting potential complications, and optimizing post-operative care to achieve the best possible outcomes for each patient [78].

Incorporating AI-powered decision support systems into surgical education and training is another important future direction [79]. AI algorithms can analyze specific surgical data, including surgical videos, case reports, and simulation data, to provide personalized feedback and guidance to trainees [80]. These systems can enhance surgical skills, improve surgical education, and support continuous professional development throughout a surgeon’s career [81].

Currently, new studies have revealed the important roles of Radiomics in SDM, which is revolutionizing the field of surgery by providing valuable insights and assisting in personalized treatment planning [82][83]. As Radiomics continues to evolve and our understanding of imaging biomarkers expands, its integration into SDM processes is promising.

Indeed, by extracting and analyzing quantitative imaging features from pre-operative images, Radiomics can provide valuable information about tumor characteristics, such as size, shape, texture, and vascularity [84]. These features can then be correlated with clinical outcomes, enabling surgeons to make more informed decisions regarding the extent of surgical resection, choice of surgical approach, and the need for adjuvant therapies [85]. In other words, Radiomics could maximize the chances of successful outcomes while minimizing unnecessary interventions [85].

In addition, another potential is the incorporation of Radiomics into surgical planning and navigation systems. Indeed, surgeons by integrating Radiomics data with pre-operative imaging, can obtain real-time and intra-operative guidance [86]. It could assist in identifying tumor boundaries, critical structures, and potential areas of tumor infiltration that may be missed with routine analysis [87]. Also, Radiomics-guided surgical navigation can enhance precision, reduce the risk of complications, and improve the overall surgical experience for both the surgeon and the patient [86][88].

Additionally, the advancement of machine learning algorithms in Radiomics provides more significant potential in predicting post-operative complications and individualizing patient management [89]. Hence, by training models using large datasets that include Radiomics features, clinical data, and post-operative outcomes, algorithms can learn to recognize patterns and make predictive models [90][91]. These models can aid in risk stratification, allowing surgeons to identify patients who may be susceptible to specific complications (e.g., post-operative infections or vascular complications) [92]. Therefore, by obtaining this information, surgeons can optimize peri-operative management and implement preventive measures to increase patient safety and improve post-operative recovery [93].

Furthermore, Radiomics can contribute to the growing field of minimally invasive surgery. Indeed, by application of imaging features derived from pre-operative images, Radiomics can help to identify patients who are suitable candidates for minimally invasive techniques, such as laparoscopic or robotic surgery [94][95]. In other words, by assessing the tumor’s characteristics and its relationship to surrounding structures, Radiomics can guide surgeons in determining the feasibility and safety of these approaches [95]. Consequently, it could potentially reduce surgical trauma, shorten recovery times, as well as improve overall patient outcomes [96].

Collaborative research efforts, advancements in technology, and ethical considerations pave the way for safer, more effective, and personalized surgical interventions [97]. Using the potential of AI, surgeons could access powerful tools and insights that can enhance decision-making, optimize surgical techniques, and ultimately improve patient outcomes, shaping the future of surgical practice [98].

## Conclusion

AI has essential roles in SDM, including its pre-operative assessments and post-operative monitoring applications. Despite challenges, AI has the potential to enhance surgical precision, reduce complications, and ultimately improve patient outcomes. As this field progressed, collaborative efforts from surgeons, researchers, and AI developers should facilitate the integration of AI into surgical practice.

## Conflicts of Interests

The authors declare that there are no conflicts of interest.
